# Optimal management of HIV- positive adults at risk for kidney disease in Nigeria (Renal Risk Reduction “R3” Trial): protocol and study design

**DOI:** 10.1186/s13063-019-3436-y

**Published:** 2019-06-10

**Authors:** Muktar H. Aliyu, Usman J. Wudil, Donna J. Ingles, Bryan E. Shepherd, Wu Gong, Baba M. Musa, Hamza Muhammad, Mahmoud U. Sani, Aliyu Abdu, Aisha M. Nalado, Akinfenwa Atanda, Aima A. Ahonkhai, Talat A. Ikizler, Cheryl A. Winkler, Jeffrey B. Kopp, Paul L. Kimmel, C. William Wester

**Affiliations:** 1 Vanderbilt Institute for Global Health (VIGH), Nashville, TN USA; 2 Department of Health Policy, Vanderbilt University Medical Center, Nashville, TN USA; 3 Department of Biostatistics, Vanderbilt University Medical Center, Nashville, TN USA; 4 Department of Medicine, Division of Nephrology, Vanderbilt University Medical Center, Nashville, TN USA; 5 Department of Medicine, Division of Infectious Diseases, Vanderbilt University Medical Center, Nashville, TN USA; 6 Department of Medicine, Aminu Kano Teaching Hospital, Kano, Nigeria; 7 Department of Pathology, Bayero University, Aminu Kano Teaching Hospital, Kano, Nigeria; 8 Basic Research Laboratory, Molecular Genetic Epidemiology Section, Frederick National Laboratory for Cancer Research sponsored by the National Cancer Institute, NIH, Frederick, MD USA; 9 National Institute of Diabetes and Digestive and Kidney Diseases, Division of Kidney, Urologic and Hematologic Diseases, NIH, Bethesda, MD USA

**Keywords:** *APOL1*, Microalbuminuria, Kidney disease, Lisinopril, HIV, Nigeria

## Abstract

**Background:**

Individuals with two copies of the apolipoprotein-1 (*APOL1)* gene risk variants are at high risk (HR) for non-diabetic kidney disease. The presence of these risk variants is highest in West Africa, specifically in Nigeria. However, there is limited availability of dialysis and kidney transplantation in Nigeria, and most individuals will die soon after developing end-stage renal disease. Blocking the renin angiotensin aldosterone system with angiotensin-converting enzyme inhibitors (ACEi) is a well-recognized strategy to slow renal disease progression in patients with diabetes mellitus with chronic kidney disease (CKD) and in patients with HIV-associated nephropathy. We propose to determine whether presence of the *APOL1* HR genotype alters or predicts responsiveness to conventional therapy to treat or prevent CKD and if addition of an ACEi to standard combination antiretroviral therapy (ART) reduces the risk of kidney complications among non-diabetic Nigerian adults.

**Methods/design:**

We will screen 2600 HIV-positive adults who have received ART to (1) determine the prevalence of *APOL1* risk variants and assess whether *APOL1* HR status correlates with prevalent albuminuria, estimated glomerular filtration rate (eGFR), and/or prevalent CKD; (2) assess, via a randomized, placebo-controlled trial (RCT) in a subset of these participants with microalbuminura (*n* = 280) whether addition of the ACEi, lisinopril, compared to standard of care, significantly reduces the incidence or progression of albuminuria; and (3) determine whether the *APOL1* HR genotype is associated with worse kidney outcomes (i.e. eGFR slope or regression of albuminuria) among participants in the RCT.

**Conclusions:**

This study will examine the increasing prevalence of kidney diseases in HIV-positive adults in a West African population, and the relationship between these diseases and the *APOL1* high-risk genotype. By evaluating the addition of an ACEi to the care of individuals with HIV infection who have albuminuria, our trial will provide definitive evidence to guide strategies for management and clinical care in this population, with the goal of reducing HIV-related kidney complications.

**Trial registration:**

ClinicalTrials.gov, NCT03201939. Registered on 26 August 2016.

**Electronic supplementary material:**

The online version of this article (10.1186/s13063-019-3436-y) contains supplementary material, which is available to authorized users.

## Background

Although antiretroviral therapy (ART) has substantially reduced the impact of HIV-associated kidney disease in the USA, the HIV epidemic continues globally, including in kidney disease-susceptible African and African-descent populations [1–3]. Chronic kidney disease (CKD) is at least threefold to fourfold more common in Africa than in developed countries [4–6]. The reported prevalence of CKD in HIV- positive ART-naïve patients ranges from 6% to 48% across sub-Saharan Africa, with the highest prevalence reported in Nigeria [5–10]. A paucity of research, however, has focused on the cause, progression, and prevention of CKD in this region [9]. This is of particular concern, given that Nigeria has the second largest number of people living with HIV (PLHIV) globally, among whom only ~ 30% are receiving ART [11, 12].

The true prevalence of HIV-associated nephropathy (HIVAN) worldwide is unknown, particularly in Africa [12]. Previous studies suggest that approximately 10% of HIV- positive persons with recent African ancestry develop HIVAN if untreated at some point in their lifetime [12–14]. As HIVAN and focal segmental glomerulosclerosis (FSGS) emerge exclusively (HIVAN) or largely (FSGS) in persons of African descent, several studies have investigated genetic associations with predisposition to CKD in these patients [15–27]. Genovese et al. described two risk alleles in the Apolipoprotein-1 (*APOL1*) gene [22], Kopp et al. and others showed that carriage of these two alleles (*APOL1* high-risk (HR) genotype) confers sizeable risk (with odds ratios ranging from 3.1 to 89) for FSGS and hypertension-attributed end-stage renal disease (ESRD) [15, 21, 22, 28]. These variants are present only on African-origin chromosomes, with frequency of these risk alleles highest in West Africa, specifically in Nigeria [29, 30]. As the *APOL1* effect is largely recessive, the ~ 25% of the West African population carrying *APOL1* HR genotypes are at substantially increased risk of kidney disease. An estimated 50% of HIV-positive, ART-untreated individuals carrying HR genotypes will develop CKD [31, 32]. Even when HIV replication is suppressed, *APOL1* HR individuals remain at greatly increased risk for FSGS and ESRD, similar to HIV-uninfected *APOL1* high-risk individuals [23].

The renin-angiotensin aldosterone system (RAAS) is a central driver of the pathophysiology of CKD [33, 34]. Kidney dysfunction can be regarded as a continuum that extends from endothelial and podocyte dysfunction to microalbuminuria, macroalbuminuria, ESRD, and ultimately premature death, with all stages associated with progressively increasing cardiovascular risk [35]. Preventing development and progression of kidney disease requires tight blood pressure control and, due to the important role of the RAAS in the pathogenesis of kidney disease, agents that inhibit this system (angiotensin-converting enzyme inhibitors (ACEi) or angiotensin receptor blockers (ARB)) are recognized first-line therapy [3, 35, 36], both effectively lowering blood pressure and directly acting on the kidney.

In this study, we are screening HIV-positive, adults who have received ART, to determine whether *APOL1* risk variants alter or predict responsiveness to RAAS inhibition, and if addition of an ACEi to standard ART reduces risk of renal and other end-organ complications within this population. This will provide the first randomized controlled trial (RCT) evidence informing the optimal strategy to manage HIV-positive adults with albuminuria, particularly those with *APOL1* risk variants. Confirmation of the rate of HIV-positive adults carrying the *APOL1* HR genotype will also have significant implications for clinical care (including monitoring strategies and personalized medicine approaches) in Nigeria and across sub-Saharan Africa, and in the Americas and Europe where descendants of sub-Saharan Africans now live.

## Methods/design

### Setting

This study will be conducted in the U.S. President’s Emergency Plan for AIDS Relief (PEPFAR)-funded HIV clinic at Aminu Kano Teaching Hospital (AKTH) in Kano, a state in northwestern Nigeria. Kano is the most populous state in Nigeria, and has an HIV prevalence of 1.3% [37]. AKTH is a large tertiary center that provides care for more than 10,000 HIV-positive adults. AKTH has longstanding collaborations with Vanderbilt University Medical Center and is the site for multiple clinical trials, primarily funded by the U.S. National Institutes of Health (NIH) and the Bill and Melinda Gates Foundation.

### Study design

The prevalence of *APOL1* risk variants will be determined. Using a recessive model for the primary analysis, correlation will be tested between *APOL1* genotypes and markers of kidney disease (microalbuminuria, estimated glomerular filtration rate (eGFR)) in 2600 HIV-positive adults. From this population, 280 adults with confirmed microalbuminuria will be enrolled into a randomized, double-blinded, placebo-controlled study to assess the effect of addition of an ACEi to standard ART. We will apply block randomization using baseline urine albumin-to-creatinine ratio (uACR) values to ensure balance of this key covariate between the randomization arms, thus minimizing bias in the classification of outcomes and potential adverse effects of ACEi therapy.

### Specific aims

The specific aims of this study are:

1. To determine the prevalence of *APOL1* renal risk variants among 2600 HIV-positive individuals in Nigeria and assess whether *APOL1* HR status correlates with prevalent albuminuria, eGFR, and/or prevalent CKD (defined as macroalbuminuria or eGFR < 60 ml/min/1.73 m^2^) in this population.
2. To assess whether RAAS inhibition with an ACEi compared to placebo, significantly reduces incidence of albuminuria or reduced eGFR among 280 HIV-positive individuals with microalbuminuria who are receiving ART.
3. To determine whether the *APOL1* HR genotype is associated with worse longitudinal kidney outcomes among HIV-positive Nigerians with prevalent albuminuria, with regard to progression of albuminuria and eGFR decline.

The primary endpoints are:

1. Regression from microalbuminuria (urine albumin-to-creatinine ratio (uACR) = 30–300 mg/g) to normoalbuminuria (uACR < 30 mg/g)
2. Progression from microalbuminuria (uACR = 30–300 mg/g) to macroalbuminuria (uACR > 300 mg/g) by study arm
3. Mean change in uACR

The secondary endpoints are:

1. Doubling of serum creatinine concentration from baseline
2. Proportion experiencing a 40% decline in eGFR
3. All-cause mortality
4. Change in clinical/performance status as ascertained via two measures, specifically the World Health Organization (WHO) Quality of Life (WHOQOL-HIV) tool [38, 39] and the Karnofsky performance score [40]

### Study population

#### Inclusion criteria for aim 1: prevalence and correlates of *APOL1* high-risk status

The inclusion criteria are:

- 18–70 years of age
- HIV-positive (based on medical chart review to confirm HIV-positive status (previous documented HIV ELISA results)
- Receiving ART for a minimum of 6 months
- Able and willing to provide written, informed consent

The eligible population of the HIV clinic at AKTH is 40% male. We anticipate that women will be more likely to enroll in this study, given our previous experience. Therefore, study staff will specifically target men for enrollment to achieve gender balance in the study population. Strategies that will be used by staff include use of male counselors in the clinic, use of male peer counselors for targeted recruitment, and extension of clinic hours to accommodate male participants who may not be able to attend due to work engagements. These strategies have all been used successfully by our team in previous studies and HIV scale-up efforts in Nigeria and Mozambique [41, 42].

#### Inclusion and exclusion criteria for aim 2: ACEi and proteinuria reduction

The inclusion criteria are:

- Participation in the study for analysis of study aim 1: prevalence of *APOL1* high-risk status and clinical correlates.
- Suppressed plasma viral load (< 20 copies/milliliter) within the past 6 months, and no unsuppressed viral load during that time. Routine viral load monitoring has only recently been implemented at AKTH. By enrolling only patients with recent evidence of viral suppression, we will reduce the impact of ongoing HIV-associated viral replication in non-adherent persons as a contributing cause of immune activation and therefore end-organ complications, including kidney disease.
- Microalbuminuria, with average uACR between 30 and 300 mg/g, based on two first morning voids, obtained 4–8 weeks apart.
- Hematuria reading ≤ 1+ on urine dipstick.
- eGFR = > 60 ml/min/1.73m^2^ (using CKD-EPI-Cr-CyC equation) [43, 44].

and:

- If female, non-pregnant (documentation of negative urine pregnancy test) and not breastfeeding/lactating. In addition, if female (with reproductive potential), needs to be reliably/consistently receiving contraceptives (including documentation of receiving reliable/consistent contraceptives; including enrolment in family planning/consultative services).

The exclusion criteria are:

- K+ > 5.0 mEq/L, or reasons to be concerned about hyperkalemia
- History of diabetes mellitus (would qualify for treatment with an ACEi/ARB) (note, standardized diabetes screening procedure (fasting or random glucose ≥ 6.1 mmol/L))
- Hematuria reading ≥ 2+ on urine dipstick (assuming they might have glomerulonephritis from chronic active hepatitis B and/or C as both conditions can also cause albuminuria/proteinuria)
- Poorly controlled hypertension (≥ 3 blood pressure (BP) readings > 160/110 mmHg in the past 6 months)
- Persistent symptomatic hypotension (BP < 90/60 mmHg) (on two or more successive readings)
- Known history of chronic congestive heart failure
- Initial screening uACR value > 300 mg/g plus urine dipstick reading ≥ 2+ for protein
- Currently receiving an ACEi and/or ARB
  or:
- Lack of suitability as a study candidate (i.e. active substance use disorder, active use of potentially nephrotoxic medication(s) (e.g. traditional medicines, etc.) and/or history of poor compliance (e.g. multiple missed scheduled clinic appointments, etc.))

### Randomization

For aim 2, comparing the addition of an ACEi with placebo, the 280 enrolled patients will be randomized by block using baseline uACR values to ensure even distribution of these values between the intervention and placebo groups. Specifically, enrolled patients will be stratified into three groups by uACR values in the range of 30–59 mg/g, 60–99 mg/g, and 100–300 mg/g. Half of the participants in each block will be randomly assigned to the intervention (lisinopril) arm and the other half to the control (placebo) arm. Study participants that become lost to follow up for any reason will not be replaced.

### Study procedures

Each consenting participant will have a full medical evaluation and will provide two first-morning-void urine specimens (5 ml each, 4–8 weeks apart) to obtain mean uACR values. Assays will be performed with the Roche Hitachi Cobas C 311 (Roche Diagnostics GmBH, Mannheim, Germany) system, using pyrogallol red urine creatinine by a kinetic Jaffe method and urine albumin by immunoturbidimetry [45, 46]. Each consenting patient will also provide one blood sample for measurement of serum creatinine and cystatin C (CyC), from which we will calculate eGFR using the CKD-EPI-Cr-CyC equation [43, 44]. During their first clinic visit, all screened patients will have their charts reviewed to confirm HIV-1-positive status, and CD4+ count and viral load testing results, per existing standard of care (SOC). Each screened patient will be given a unique study ID number, which the study team will maintain. The study ID can be linked back to individual patients and will therefore be kept in a secure location. All information linking patients to ID numbers will be maintained in a secure location after the completion of aims 1–3 so that long-term follow-up (even at a reduced frequency) will be possible pending the team securing additional/separate funding. However, once all funded analyses have been completed, all information linking patients to ID numbers will be destroyed. Individual patient data that will be collected and maintained by de-identified study ID include age, race/ethnicity, sex, weight, body mass index (BMI), and comorbid medical conditions such as prior/current opportunistic infections, syphilis, cancer, hypertension, and other cardiovascular diseases.

### Nigerian standard of care for HIV disease

The Nigeria HIV/AIDS adult treatment guidelines recommend assessment of all HIV-positive patients at first enrollment in the HIV clinic to include a complete history and physical examination, clinical and immunological staging of disease, and review of laboratory results. In addition, evaluation of nutritional and psychosocial status, assessment of readiness for therapy, and development of patient-specific adherence strategy is recommended for all patients. All HIV-positive patients, regardless of CD4+ cell count, are eligible to start ART. The first-line regimens include tenofovir (TDF) + lamivudine (3TC) + efavirenz (EFV); TDF + 3TC + nevirapine (NVP); zidovudine (AZT) + 3TC (or emtricitabine (FTC)) + EFV; and AZT + 3TC (or FTC) + NVP. A boosted protease inhibitor plus two nucleoside reverse transcriptase inhibitors (NRTIs) are recommended for second-line ART. Available protease inhibitors include ritonavir-boosted lopinavir and atazanavir. The government of Nigeria will transition to tenofovir, lamuvidine, plus the integrase strand transfer inhibitor (INSTI) dolutegravir (TLD) during 2018–2019. As a result, all HIV-positive persons (> 10 years of age and weighing > 30 kg) will be switched to TLD during this transition period. In patients with renal insufficiency (i.e. a documented increase in serum creatinine concentration), the guidelines recommend ART regimen dosage modifications if the calculated creatinine clearance (CrCl) is reduced at baseline (i.e., TLD will not be initiated in adult patients with a calculated eGFR of < 50 mL/min/1.73m^2^ or if their CrCl decreases significantly during follow up to < 50 mL/min/1.73m^2^ from their baseline values). Modifications include switching patients from TDF to abacavir and investigating potential causes of their worsening renal function. Patients with reductions in CrCl should undergo an evaluation for potential causes of decreased renal function and have serum Cr concentration monitored more frequently until resolution of renal insufficiency or failure. Adjustments to drug dosage should be based on the recommendations of the drug manufacturers. The national treatment guidelines recommend that routine follow-up assessment of HIV-positive patients should cover signs/symptoms of HIV-related conditions and potential medication toxicities, adherence, response to therapy, weight, and laboratory monitoring. Every 3 months, the patient should undergo a physical exam and clinical screening for tuberculosis (TB). Every 6 months, the following tests should be performed: CD4+ cell count, viral load testing, hemoglobin (Hgb) and hematocrit (Hct), alanine aminotransferase (ALT), and calculated CrCl. Aspartate aminotransferase (AST), alkaline phosphatase (alk phos), fasting blood glucose (FBG), glycosylated hemoglobin (Hgb A1C), amylase, urine pregnancy testing, lipid profiles, serum electrolyte concentrations, sputum for acid fast bacilli (AFB, using conventional microscopy and/or GeneXpert MTB/RIF technology), and chest radiography will be performed as clinically indicated.

### Genetic specimen collection and analysis

We will collect peripheral blood in one 10-ml EDTA vacutainer tube, from all patients included in the analysis for aim 1. Blood will be processed for buffy coat and plasma, aliquoted into multiple vials, and stored at − 80 °C. Specimens will be stored and shipped in batches to the NIH for DNA extraction (Qiagen QIAamp DNA Mini Kit (Germantown, MD)) and genetic testing for *APOL1* risk variants. Genotyping will be performed using the following TaqMan custom assay IDs: [G1] AH20SD1, rs73885319 and AHWR1JA, rs60910145; [G2] AH1RT7T, rs71785313 (San Diego, CA, USA) targeting the three chromosome 22 *APOL1* variants associated with CKD and HIV-associated nephropathy. We will define the *APOL1* genotype from the number of risk alleles. In recessive models, individuals exhibiting two risk alleles (G1/G1, G1/G2, or G2/G2) will be assigned to the high-risk (HR) group, while individuals carrying no or one risk allele (G0/G0, G0/G1, and G0/G2) will be assigned to the low-risk (LR) group. We will also perform exploratory analyses to examine the number of risk alleles (0, 1, or 2 alleles) to determine whether the association between *APOL1* genotype and the risk of events is additive.

### Longitudinal study assessments

All patients enrolled in the RCT (for analysis of study aim 2) will undergo the following tests at baseline (time of randomization) and every 3 months while on study for 2 years of follow up: serum potassium [K+], CyC concentration, serum creatinine concentration, and uACR. Clinical performance status (as measured by the WHOQOL-HIV tool and the Karnofsky performance score) will be evaluated at baseline, 12, and 24 months. This is in addition to routine standard laboratory monitoring according to existing national Nigeria HIV care and treatment guidelines; specifically, serum chemistry analysis, CD4+ cell count, viral load, liver function tests, and lipid profile monitoring at least every 6 months.

### Study medication and adherence during study phases

#### Medication initiation

Participants in the intervention arm will be given lisinopril at a starting dose of 10 mg/day at the time of enrollment for the analysis of aim 2. Participants in the control arm will receive a matched placebo. Participants who are found to have symptomatic hypotension and/or new onset/persistent grade 3 (or higher) hyperkalemia after taking study medication (as evaluated at day 3 (safety phone call), week 1, month 1, or during an unscheduled visit) will be removed from the study.

#### Medication up-titration

Participants who tolerate the initial study dose will have their dose increased to 20 mg/day lisinopril (or matched placebo) at their 1-month study visit. After dose escalation, all participants will undergo safety monitoring in the form of a safety phone call at day 3 and study visits for blood draw/safety evaluation at week 1 and month 1 after dose escalation. Those who do not tolerate an increase to the 20 mg/day dose will be returned to the 10 mg/day dose, while those who are able to tolerate an increase to the 20 mg/day dose will be closely monitored for adverse events and reassessed at their 3-month study visit for a possible dose escalation to 40 mg/day.

Participants who tolerate the lisinopril 20 mg/day dose will have their dose increased to 40 mg/day at their 3-month study visit. All study participants having a dose escalation from 20 to 40 mg/day active medication (or matched placebo) will undergo the same safety monitoring procedures as above at day 3, week 1, and month 1. Those who do not tolerate an increase to the 40 mg/day dose will be returned to the 20 mg/day dose, while those who are able to tolerate an increase to the 40 mg/day dose will remain at this dose for the duration of the study.

#### Alternative medication

**Fig. 1:**
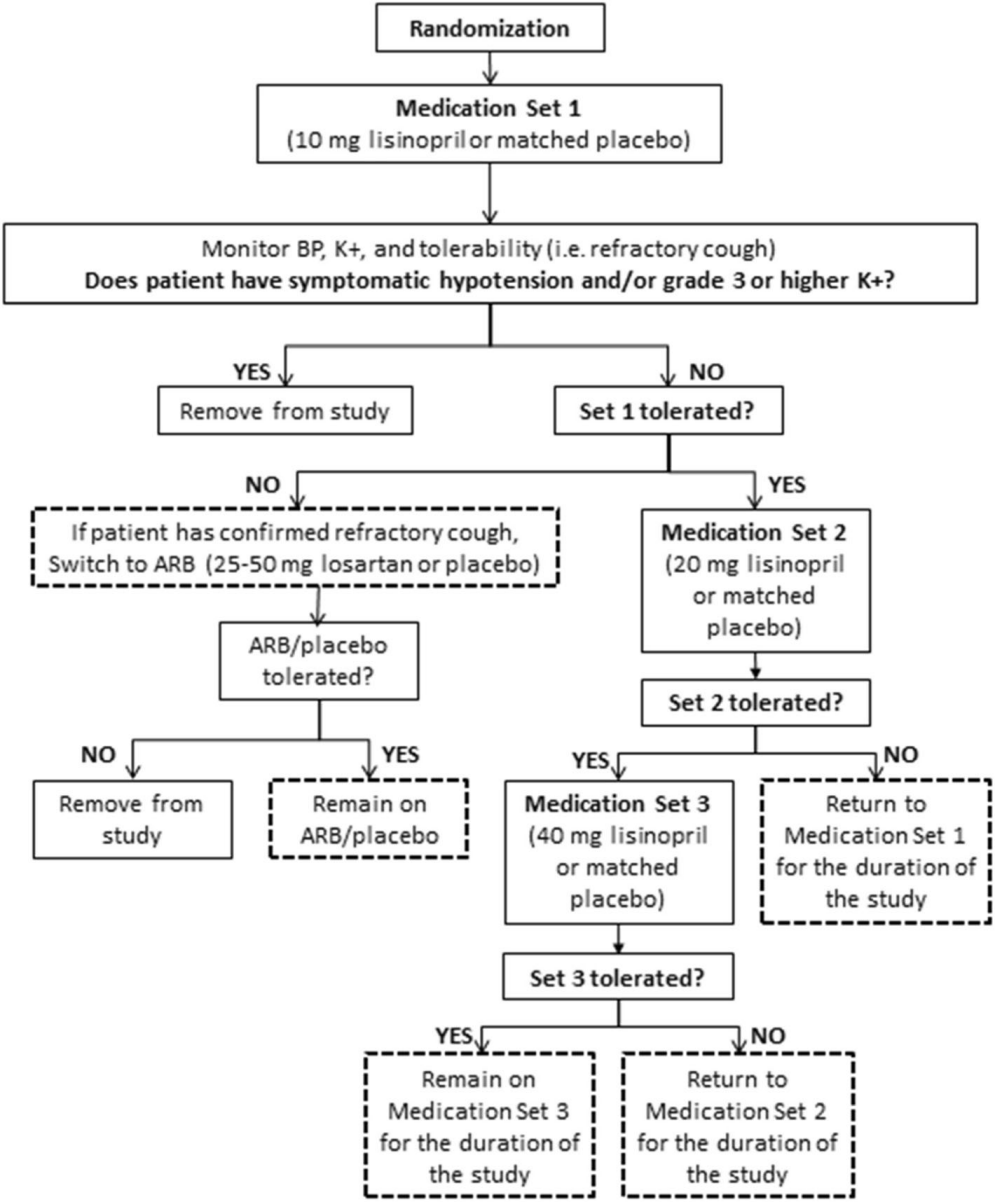
Flow diagram of study medication dosing. Endpoints are outlined in dotted boxes. Symptomatic hypotension will be defined as a reading lower than 90 mm Hg systolic or 60 mm Hg diastolic pressure, with associated symptoms (i.e. feeling of lightheadedness, weakness). All low blood pressure (BP) readings will be confirmed, orthostatic blood pressure will be obtained (when clinically indicated/appropriate), and all readings will be put in context of the individual’s normal blood pressure readings. Patients removed from the study due to failure to tolerate either the primary (lisinopril) or alternative (losartan) study drug will be unblinded to enable their physicians to best treat any conditions. Hyperkalemia will be graded using the established DAIDS toxicity grading scale (Version 2.1, July 2017); with grade 3 hyperkalemia = 6.0–6.5 mmol/L and grade 4 hyperkalemia = 6.5–7.0 mmol/L. ARB, angiotensin receptor blockers

Participants who develop problematic adverse events that are plausibly attributable to lisinopril (particularly confirmed refractory cough) at any time during the study will be transitioned to the equivalent dose of an ARB (losartan), at a dose of 25 or 50 mg/day (depending on the dose of lisinopril or matched placebo they were receiving at the time of their adverse event). The treatment allocation of participants in need of antihypertensive treatment, who develop a refractory cough while on study medication, will be unblinded due to the severity of this adverse event, so it can be listed on the patient’s active list of drug intolerances and so they will not be prescribed ACE inhibitors in the future. Participants who do not tolerate either study medication (lisinopril or losartan) will be removed from the study and referred for further care if needed (Fig. 1). Any study participant who develops allergic/cutaneous hypersensitivity skin reaction of grade ≥ 3, deemed “definitely” or “possibly related to study medication” at any time during the study, will immediately be removed from the study and referred for care if needed (note, study clinicians in consultation with the study oversight and safety committee members may also discontinue study medication (and unblind the participant) for any grade 2 allergic/hypersensitivity reaction (if deemed that it could be an early clinical manifestation of angioneurotic edema) on a case-by-case basis, to ensure study participant safety).

**Fig. 2:**
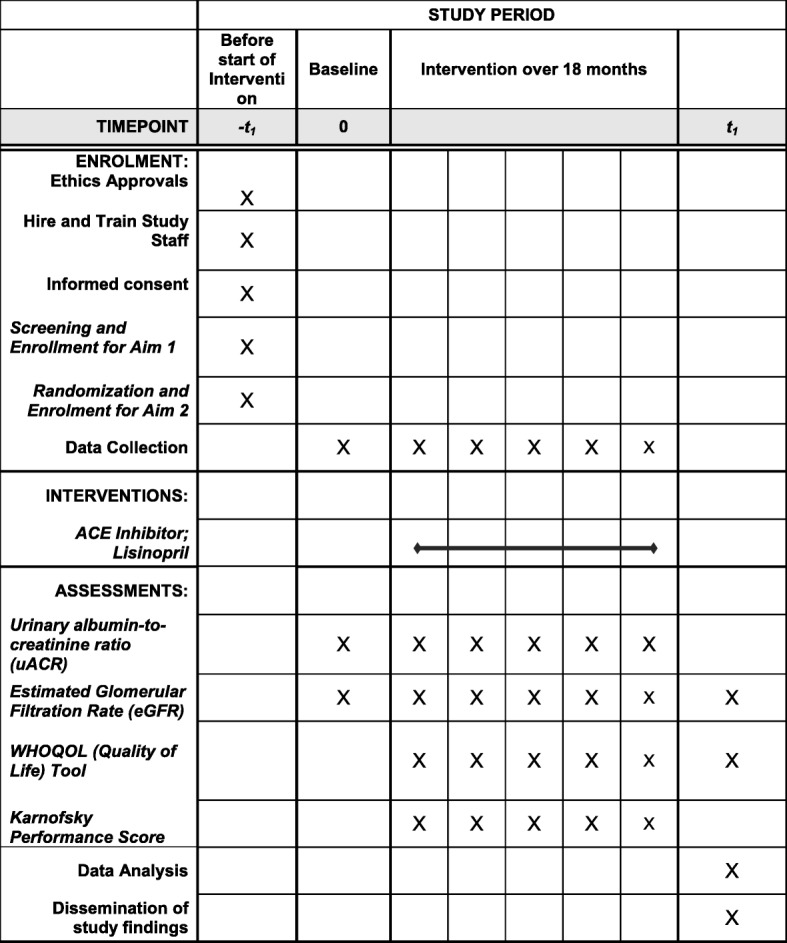
SPIRIT Figure

### Adherence

We will ascertain adherence to study medication by self-report, medication possession ratio (using pharmacy prescription/dispensing data), and by measuring change in blood pressure (post hoc) among patients randomized to the intervention arm. Using evidence-based strategies as per standard of care [47–49], we will implement educational and behavioral interventions for persons categorized as having poor adherence. For persons demonstrating average to high adherence, we will implement positive reinforcement with the continuation of educational interventions [47] throughout the study [47, 50]. Please refer to Fig. 2 (SPIRIT Figure) for study procedures/specifics.

### Power considerations

#### Sample size for aim 1: prevalence and correlates of *APOL1* high risk status

Given the strict inclusion criteria for aim 2, a screening sample size of 2600 participants is needed for the analysis of aim 1, to yield 280 participants for enrollment and randomization for the analysis of aim 2. With 2600 persons screened for aim 1, the prevalence of *APOL1* renal risk variants may be estimated with precision (half-length of the 95% confidence interval) equal to 0.02 or better. Assuming a microalbuminuria prevalence of 17.5%, based on recent data obtained from HIV-infected adults (*n* = 460) screened at AKTH [51], we will have 90% power to detect a relative risk of microalbuminuria of 1.37 or higher in participants with *APOL1* renal risk variants compared to those without. We anticipate that 25% of participants will have two *APOL1* risk variants.

#### Sample size for aim 2: ACE inhibitor and proteinuria reduction

We will enroll 280 eligible participants from aim 1 for 1:1 randomization in the aim-2 trial. Based on our prior experience with ongoing RCTs at this study location, we anticipate that enrollment for aim 2 will take 12 months, and there will be a maximum of 10% loss to follow up at the end of year 2, yielding 252 patients on study (126 per arm). At 2 years, we hypothesize that a larger proportion of intervention participants will regress from microalbuminuria to normoalbuminuria (uACR < 30 mg/g) with a hazard ratio of 2.50. Using the log rank test, we will have 80% power if the rate of regression in the placebo arm is at least 12%, and 90% power if that rate is 16% or higher with a 1.7% type I error [52] (a type I error rate of 1.7% is used to achieve a Bonferroni-adjusted family-wise error rate of 5%, given that we have three primary endpoints). We also hypothesize that at 2 years, a larger proportion of patients in the placebo arm will progress from microalbuminuria to macroalbuminuria (uACR > 300 mg/g). With 126 patients per arm, we will have > 95% power with a 1.7% type I error to detect a difference in progression from microalbuminuria to macroalbuminuria of 60% in the placebo arm compared to 25% in the intervention arm (hazard ratio ≈ 0.42).

#### Sample size for aim 3: impact of *APOL1* status on long-term renal outcomes (i.e. eGFR slope or regression of albuminuria) among participants in the RCT

Assuming 30% (*n* = 84) of aim 2 study participants have the high-risk genotype and the rate of regression is similar to that hypothesized in aim 2, we anticipate having 80% power to detect hazard ratios on the order of 2.4–3.2. Specifically, if the rate of regression is 22% (e.g. 10% and 25% in the control and intervention arms, respectively), we will have 80% power to detect a hazard ratio of 3.2 between the low-risk and high-risk genotypes. If the rate of regression is 35% (e.g. 20% and 40% in the control and intervention arms, respectively), we will have 80% power to detect a hazard ratio of 2.4. With regard to progression, if the overall event rate is 42.5%, we anticipate having 80% power to detect a hazard ratio of 2.5 between the high and low risk genotypes. If the overall rate of progression is 28%, we anticipate approximately 80% power to detect a hazard ratio of 2.5.

### Statistical considerations

#### Statistical analysis for aim 1: prevalence and correlates of *APOL1* high-risk status

Based on previous genetic association studies in HIVAN [15, 20–32], we will test the recessive genetic models for *APOL1* HR genotypes with microalbuminuria and CKD. We will test the additive model (2 vs 1 vs 0 variants) in a secondary analysis. We will estimate the prevalence and associated 95% confidence intervals of *APOL1* renal risk variants. We will test whether the presence of these variants is associated with log_10_ uACR or eGFR using the Wilcoxon rank sum test. Multivariable linear regression will be used for the additive model and to adjust for potential confounders, including sex, age, CD4+ cell count, viral load, and the presence/absence of specific comorbid medical conditions (chiefly hypertension, cardiovascular disease, and diabetes mellitus). For linear regression, dependent variables will be transformed as appropriate to satisfy modelling assumptions. To relax linearity assumptions, covariates will be modelled using restricted cubic splines [53]. Modified Poisson regression, adjusted for similar variables, will be used to assess associations with CKD, microalbuminuria, and eGFR (< 60 ml/min/1.73m^2^) with 95% confidence intervals.

#### Statistical analysis for aim 2: ACE inhibitor and proteinuria reduction

We will test the hypotheses that rates of regression from microalbuminuria and progression to macroalbuminuria are different between treatment arms, using multivariable Cox regression models with the intervention variable as the primary predictor variable, adjusting for baseline (pre-randomization) covariates, including uACR, number of *APOL1* renal risk variants, CD4+ cell count, and the presence/absence of comorbid medical conditions (hypertension, cardiovascular disease). To relax linearity assumptions, baseline uACR will be included in models using restricted cubic splines [53]. Primary analyses will be on an intention-to-treat basis, although we also plan to perform per-protocol analyses. We will test the impact of ACEi use on uACR, using a linear mixed effects model to account for within-patient correlations. Of note, uACR will be measured in all participants every 3 months while on study. However, given that all participants will undergo dose titration (with active study medication versus matched placebo) for up to the first 6 months in an attempt to maximize their ACEi dosing while balancing tolerability and safety, data for the primary endpoints in this study will be obtained for each patient at 12 and 24 months after attaining the maximum tolerated dose of study medication. The model will also adjust for the baseline covariates given above. For this analysis, we anticipate log-transforming uACR to meet modeling assumptions, although other transformations will be considered if needed. Analyses for secondary endpoints will be similar to those described above. Time-to-event outcomes (doubling of serum creatinine concentration from baseline, all-cause mortality, and 40% decline in eGFR) will be assessed using multivariable Cox regression models. Results will be displayed using Kaplan-Meier estimates. Continuous outcomes (eGFR, quality of life metrics, and blood pressure) will be analyzed using mixed effects models after proper transformation of the outcome variable, if necessary. Baseline covariates listed above will be included in all secondary analyses.

#### Statistical analysis for aim 3: impact of *APOL1* status on long-term renal outcomes

Analyses for aim 3 will be the same as those outlined in aim 2, except the predictor of interest will be *APOL1* haplotype. We will fit recessive models (2 copies of *APOL1* risk variants versus 0–1 copies) and additive models (number of copies of *APOL1* risk variants). Analyses will be adjusted for intervention arm. Please refer to Additional files 1 and 2, namely the SPIRIT 2013 Checklist: Recommended items to address in a clinical trial (Additional file 1) and the CONSORT 2010 Checklist of information to include when reporting a RCT (Additional file 2).

## Discussion

This study is innovative for a number of reasons. First, microalbuminuria among HIV-positive adults in Nigeria most likely represents underlying early structural kidney disease. This was the case in a small kidney biopsy series performed among HIV-positive adults presenting with persistent microalbuminuria in South Africa, of whom six of seven patients were found to have HIV-associated nephropathy. [12] The present study will be the first to evaluate therapy to treat microalbuminuria in this population with the goal of preventing the progression of kidney disease.

Second, *APOL1* variants are a major driver of kidney disease in HIV-positive persons. Most existing data on *APOL1* risk variant prevalence are based on DNA from studies with very small sample sizes among specific African ethnic groups. This will be the largest genetic risk variant survey to date among individuals in West Africa, the region of the world with the highest documented carriage rates of the *APOL1* high-risk (HR) genotype.

Third, if early therapy can reduce albuminuria, it could also reduce the burden of HIV-associated CKD and associated morbidity and premature mortality. Such findings would make a strong case for the adoption of population-based screening for albuminuria using uACR measurements (compared to less accurate urine dipstick and cumbersome 24-h urine protein measurements). This testing would also greatly assist in the identification of persons in need of early intervention. In resource-poor settings, where access to therapies for ESRD (dialysis and kidney transplantation) are not widely available, prevention of CKD may prolong life.

Fourth, determining if the presence of an *APOL1* risk genotype correlates with ESRD risk (microalbuminuria, reduced eGFR, and/or early CKD), and determining whether it influences longitudinal renal outcomes among patients with prevalent albuminuria, is novel and will inform treatment guidelines and result in significant public health benefits, particularly if our results show that affordable and readily available treatment can be instituted early in the disease continuum.

Finally, various interventional studies involving small numbers of subjects have been performed showing that the provision of RAAS inhibition is safe and may be beneficial when given to HIV-positive adults with HIV-associated nephropathy manifesting with varying levels of proteinuria [36, 54–59]. Despite a strong rationale and encouraging preliminary data, the role of RAAS inhibition in HIV-positive subjects with or at-risk for HIV-associated kidney disease remains to be confirmed by well-designed and adequately powered randomized controlled trials. The R3 study as designed is well-poised to be the first RCT to provide definitive evidence on the role that RAAS inhibition as adjunctive therapy to standard ART could play in the large numbers of HIV-positive adults at risk for long-term kidney complications. Such guidance is especially relevant in West Africa where the prevalence of the *APOL1* high-risk genotype is highest globally. In addition, the promising pharmaceutical approach of the R3 study is particularly important in sub-Saharan Africa, where treatment options for ESRD (i.e. dialysis and transplantation) are extremely limited.

## Conclusions

In summary, this study will examine the increasing prevalence of kidney diseases in HIV-positive adults in a West African population, and the relationship between these diseases and the *APOL1* high-risk genotype. By evaluating the addition of an ACEi to care of individuals with HIV infection who have albuminuria, our trial will provide definitive evidence to guide strategies for management and clinical care in this population, with the goal of reducing HIV-related kidney complications.

## Additional files

Additional file 1: SPIRIT 2013 Checklist: Recommended items to address in a clinical trial protocol and related documents. (DOC 122 kb)

Additional file 2: CONSORT 2010 checklist of information to include when reporting a randomized trial. (DOC 218 kb)

## Data Availability

Not applicable.

## Abbreviations

ACEi: Angiotensin converting enzyme inhibitor
AKTH: Aminu Kano Teaching Hospital; Kano, Nigeria-study site
*APOL1 HR*: Apolipoprotein-1 high-risk genotype
*APOL1*: Apolipoprotein-1
ARB: Angiotensin receptor blocker
ART: Combination antiretroviral therapy
CKD: Chronic kidney disease
CKD-EPI: Chronic kidney disease epidemiology collaboration
Cr: Serum creatinine
CrCl: Calculated creatinine clearance
CyC: Serum cystatin C
eGFR: Estimated glomerular filtration rate
ELISA: Enzyme-linked immunosorbent assay
ESRD: End-stage renal disease
FSGS: Focal segmental glomerulosclerosis
HIV: Human immunodeficiency virus
HIVAN: HIV-associated nephropathy
HR: High risk
INSTI: Integrase strand transfer inhibitor
PEPFAR: President’s Emergency Plan for AIDS Relief
PLHIV: People living with HIV
R3: Renal Risk Reduction study
RAAS: Renin-angiotensin aldosterone system
RCT: Randomized controlled trial
TLD: Tenofovir (TDF), lamuvidine (3TC), plus dolutegravir (DTG)
uACR: Urine albumin-to-creatinine ratio
WHOQOL-HIV: World Health Organization Quality of Life HIV instrument
